# CTX (Crosslaps) Rather than Osteopontin Is Associated with Disturbed Glucose Metabolism in Gestational Diabetes

**DOI:** 10.1371/journal.pone.0040947

**Published:** 2012-07-23

**Authors:** Yvonne Winhofer, Florian W. Kiefer, Ammon Handisurya, Andrea Tura, Katharina Klein, Barbara Schneider, Rodrig Marculescu, Oswald F. Wagner, Giovanni Pacini, Anton Luger, Thomas M. Stulnig, Alexandra Kautzky-Willer

**Affiliations:** 1 Division of Endocrinology and Metabolism, Department of Internal Medicine III, Medical University of Vienna, Vienna, Austria; 2 Division of Nephrology, Department of Internal Medicine III, Medical University of Vienna, Vienna, Austria; 3 Metabolic Unit, Institute of Biomedical Engineering, National Research Council, Padova, Italy; 4 Department of Obstetrics and Gynecology, Medical University of Vienna, Vienna, Austria; 5 CeMSIIS, Section of Medical Statistics, Medical University of Vienna, Vienna, Austria; 6 Clinical Institute for Medical and Chemical Laboratory Diagnostics, General Hospital of Vienna, Vienna, Austria; 7 Christian Doppler-Laboratory for Cardio-Metabolic Immunotherapy, Medical University of Vienna, Vienna, Austria; Virgen Macarena University Hospital, Spain

## Abstract

**Objective:**

Reciprocal interaction between bone and glucose metabolism might play a pivotal role in the development of type 2 diabetes. We recently demonstrated that osteocalcin is increased in women with gestational diabetes (GDM) compared to healthy pregnant women and related to enhanced insulin secretion. Here, we aimed to investigate the role of the bone resorption marker CTX and osteopontin (OPN), a key molecule in subclinical inflammation underlying insulin resistance, in gestational diabetes.

**Methods:**

Insulin sensitivity and secretion (derived from OGTT) as well as CTX and osteopontin were investigated in 26 GDM and 52 women with normal glucose tolerance during pregnancy [CON] between 24th and 28th gestational weeks; 24 women also underwent postpartum examination.

**Results:**

CTX was significantly higher in GDM compared to CON (0.44±0.20 vs.0.28±0.12 ng/ml, p<.0001) and positively correlated with osteocalcin (R = 0.64, p<.0001) and parameters of insulin secretion. Osteopontin plasma concentrations were decreased in GDM compared to CON (28.81±22.12 vs.37.68±19.63 ng/ml, p = 0.04), and did not show any relation to insulin secretion or sensitivity, but were significantly correlated with CRP (R = 0.3, p<0.007) and liver enzymes. Twelve weeks after delivery CTX and OPN were increased compared to pregnancy (both p<.0001) and did not differ between GDM and CON.

**Conclusion:**

Our findings support the idea of a tight regulation between bone and glucose metabolism, and suggest, that less curbed CTX during pregnancy might be involved in osteocalcin-mediated amelioration of insulin secretion in GDM. On the other hand, osteopontin was unrelated to insulin resistance in GDM, but associated with inflammatory markers and liver enzymes in all women.

## Introduction

In the past, bone was considered as the tissue bearing the mechanical burden of increasing body weight in our population. Nowadays, it becomes more and more obvious that bone might also be involved in the metabolic consequences which result from obesity, i.e. diabetes [1]. Hence, increasing interest emerges on the reciprocal and regulatory interaction between bone and glucose metabolism and its role in metabolic diseases.

We recently demonstrated that osteocalcin, a marker of bone remodeling, is higher in women with gestational diabetes (GDM) compared to women with normal glucose tolerance during pregnancy [CON] and related to enhanced insulin secretion in these women [2]. In consideration of current evidence from animal models, which demonstrated a supportive role of osteocalcin on beta-cell proliferation and insulin secretion [3], [4], we concluded that osteocalcin might be able to enhance insulin secretion in periods of increased insulin demand, such as a GDM-pregnancy. Hence, we postulated a compensatory role of bone for disturbed glucose metabolism in gestational diabetes.

Consequently, interest occurred whether further hormones/metabolites initially detected in bone metabolism might also contribute to the regulation of glucose metabolism in GDM.

The C-terminal cross-linking telopeptide of Type-I collagen, also termed CTX or crosslaps, is a marker of bone resorption and applied for monitoring the therapeutic effect of osteoporosis therapy in daily clinical practice. Recent investigations have postulated an important role of bone resorption in the regulation of glucose homeostasis [5]. These studies provided evidence that insulin signaling in osteoblasts decreases osteoprotegerin-expression, leading to increased bone resorption and consequent acidification of the extra-cellular matrix; this milieu at low pH is essential for decarboxylation and thus activation of osteocalcin [6]. Hence it was shown that bone resorption is essential for the activation of osteocalcin. Assuming these findings can be translated to humans, changes in osteocalcin might be accompanied by changes in CTX.

Osteopontin (OPN), on the other hand, is a multifunctional glycoprotein expressed in different cell types, e.g. osteoclasts, and is known to be involved in physiological and pathological bone mineralization but also in in many inflammatory disorders like atherosclerosis and rheumatoid arthritis [7]–[9]. Recently OPN was found to promote obesity-associated insulin resistance and hepatic steatosis. Given that late pregnancy is an insulin-resistant state and that women with gestational diabetes are known to have even increased insulin resistance, osteopontin might be implicated in the development of insulin resistance during pregnancy *per se* and/or the aggravation of insulin resistance in gestational diabetes. Of note, osteopontin was reported to be elevated in women with preeclampsia [10] and like gestational diabetes, also preeclampsia has been linked to increased insulin resistance during pregnancy [11].

Therefore, plasma concentrations of both, CTX and osteopontin, might be different in women with gestational diabetes compared to healthy controls and highlight that bone is implicated in metabolic changes during a GDM-pregnancy.

Since no data exist on CTX and osteopontin in gestational diabetes, we aimed to investigate their plasma concentrations as well as their relations to insulin sensitivity and insulin secretion in women with gestational diabetes and women with normal glucose tolerance during pregnancy and postpartum.

## Study Population and Methods

The study was performed at the outpatient’s clinic of the Department of Internal Medicine III, Division of Endocrinology and Metabolism of the Medical University of Vienna. In a previous study, described in [2], we have studied 26 women with gestational diabetes (GDM) and 52 women with normal glucose tolerance during pregnancy [CON] according to routine screening for gestational glucose intolerance between 24–28th gestational weeks; thus no woman was on treatment at the time of the examination. After an overnight fast for at least 12 hours, all women underwent a 75 g 2-hour oral glucose tolerance test (OGTT) with measurements of glucose, insulin and C peptide at timed intervals. Here, we present the same subjects, in whom CTX and OPN were additionally investigated. Hence, part of the baseline characteristics presented in Table 1 (age, BMI, parity, HbA1C) have already been published in [2].

**Table 1:** Baseline characteristics and metabolic parameters between 24–28th gestational weeks in women with gestational diabetes (GDM) and healthy controls [CON] [4] (4) (4) (4).

	GDM	CON	p-value
**n**	26	52	
**Age (years)**	33.0±6.1	32.2±6.3	ns
**Body Mass** **index (kg/m^2^)**	27.8±4.8	28.0±5.1	ns
**Parity**	1.2±1.3	0.75±1.2	ns
**CTX (ng/ml)**	0.44±0.20	0.28±0.12	<.0001
**Osteopontin** **(ng/ml)**	28.81±22.1	37.68±19.63	0.04
**HbA1C (%)**	5.0±0.4	4.7±0.5	<0.03
**Progesteron** **(ng/ml)**	94.2±48.3	84.4±49.3	ns
**Estrogen (pg/ml)**	11262.9±4360.6	11047.9±5989.2	ns
**AST (U/L)**	21.3±5.6	21.0±4.7	ns
**ALT (U/L)**	16.7±9.3	16.3±6.6	ns
**CRP (mg/dl)**	1.3±3.2	0.7±0.6	ns

24 women, 14 with prior gestational diabetes (pGDM) and 10 with normal glucose tolerance during pregnancy (pCON), underwent re-examination 12 weeks after delivery (as recommend for all women with gestational diabetes).

The protocol was approved by the human ethics committee of the Medical University of Vienna (EK-no: 771/2008) and all women gave written informed consent.

### Plasma Metabolites

CTX: The C-terminal cross-linking telopeptide of Type-I collagen was measured with ECLIA (Roche Diagnostics) with a sensitivity of 0.01 ng/ml and an interassay coefficient of variation of 4% [12], [13]. Osteopontin was measured applying ELISA (Quantinine Human Osteopontin ELISA, R&D Systems) with a CV of 2.9–4% and a mean MDD of 0.011 ng/ml; minimum concentration detectable was 7.825 ng/ml [14]. Insulin (Serono Diagnostics, Freiburg, Germany) and C-Peptide (CIS Bio International, France) were determined in duplicate by commercially available radioimmunoassay kits with interassay coefficients of variation of <5%. High-sensitive CRP was measured by means of particle-enhanced immunonephelometry (N High Sensitivity CRP Reagent, BN™ Systems, Dade Behring, Deerfield, IL). Glucose, total cholesterol [15], HDL- and LDL-cholesterol, triglycerides and HbA1C [16] were assessed by established methods in the central lab of the Medical University of Vienna.

### Data Analysis

Insulin sensitivity derived from OGTT was evaluated by OGIS, which describes glucose clearance per unit change of insulin concentration [17]. Insulin hepatic extraction and beta-cell function as the total insulin secretion from C-peptide [TIS] were assessed by mathematical modeling [18]. Insulin AUC was calculated with the trapezoidal rule. The ability of the beta cell to adapt insulin secretion to the prevailing insulin resistance was quantified by the products: OGIS × dynamic AUC insulin and OGIS × dynamic TIS. These are sometimes called disposition and adaptation indices, respectively.

### Statistical Analysis

Comparisons of quantitative variables among groups were performed using ANOVA. Since the minimum concentration of osteopontin detectable was 7.825 ng/ml (23% in GDM and 13% in CON), values below were truncated to this value and a non-parametric ANOVA (Wilcoxon) was additionally performed. Regression analyses were performed with osteopontin and CTX as dependent, and parameters of glucose metabolism as independent variables. The strength of associations was shown by correlation analyses, described by Pearson’s correlation coefficient, and grouped analyses were performed to correct for heterogeneity between the groups (GDM, CON). SAS® software (Enterprise Guide 4.3) was used for all computations. A p-value of <0.05 was considered as statistically significant.

## Results

### Increased CTX and Decreased Osteopontin Plasma Concentrations in GDM Compared to Healthy Controls

**Figure 1:**
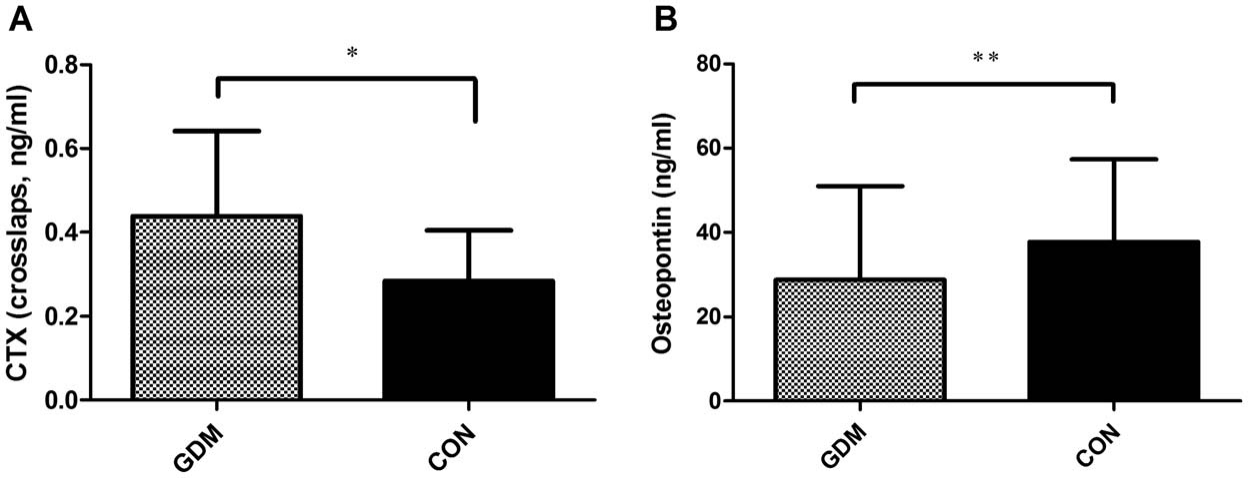
Plasma concentrations of CTX (A) and osteopontin (B) in women with gestational diabetes (GDM) and women with normal glucose tolerance during pregnancy (CON, black boxes) between 24–28th gestational weeks. *p<0001, **p = 0.04.

Plasma concentrations of CTX were significantly increased in GDM compared to CON (**Fig. 1A**) between 24–28th gestational weeks. In contrast, osteopontin (OPN) was found decreased in GDM compared to CON (**Fig. 1B**). Data and baseline characteristics are presented in **Table 1**.

### Correlation Analyses

**Figure 2:**
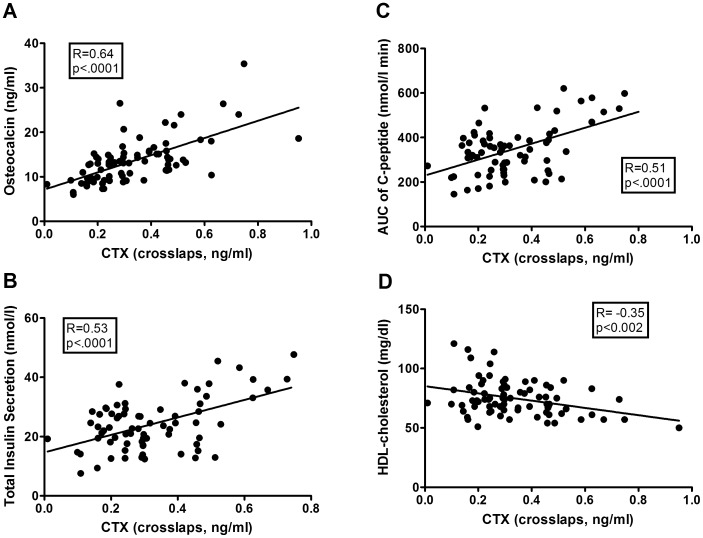
Correlation analysis in the whole study group. CTX was positively correlated with osteocalcin (A), total insulin secretion (B) and AUC of C-peptide (C) during OGTT and inversely associated with HDL-cholesterol (D).

Plasma concentrations of CTX were tightly associated with those of osteocalcin (see **Fig. 2A**) and furthermore with the dynamic AUC of glucose (R = 0.41, p = 0.0003) during OGTT. In addition, CTX was positively correlated with basal and total insulin secretion (TIS, see **Fig. 2B**) and the AUC of C-peptide (see **Fig. 2C**), which was also represented by a tight association between CTX and the disposition index (R = 0.50, p<.0001). Inverse associations were observed between CTX and HDL-cholesterol (see **Fig. 2D**), hepatic insulin extraction (R = −0.35, p<0.003) and insulin sensitivity (OGIS: R = −0.34, p = 0.003). In stepwise regression analyses, osteocalcin and TIS remained significantly associated with CTX. Of note, neither fasting insulin nor fasting glucose was correlated with CTX.

In grouped analyses, the correlations between CTX and osteocalcin as well as parameters of insulin secretion (TIS, AUC of C-peptide and the disposition index) were significant in each group, while the association between CTX and the dynamic AUC of glucose and HDL-cholesterol only remained significant in the GDM-group.

Osteopontin, on the other hand, did not show any association with parameters of insulin secretion nor insulin sensitivity. However, osteopontin was positively correlated with serum concentrations of progesterone, estrogen, liver enzymes (asparate aminotransferase [AST] and alanine aminotransferase [ALT]) and C-reactive protein and inversely associated with the AUC of glucose under dynamic conditions (see **Table 2**). In multivariable regression analyses ALT, CRP and the dynamic AUC of glucose remained significantly associated with osteopontin.

**Table 2:** Correlation analyses of osteopontin during pregnancy in the whole study group.

	Osteopontin	
	R	p-value
		
Progesterone	0.31	0.005
Estrogen	0.29	<0.01
AST (asparat-aminotransferase)	0.31	0.007
ALT (alanine-aminotransferase)	0.35	<0.002
CRP (C-reactive protein)	0.30	<0.007
High-sensitive CRP	0.26	<0.03
Dynamic AUC of glucose	0.24	0.04

R presents Pearsons’s correlations coefficient.

No correlations were observed between HbA1C and markers of bone metabolism. In grouped analyses, the association between osteopontin and progesterone as well as alanine aminotransferase (ALT) remained significant in both, the GDM and the CON group, while the association between osteopontin and estrogen only remained significant in CON.

Of note, neither female sex hormones (progesterone, estrogen), nor liver enzymes showed a correlation with insulin sensitivity (OGIS) in the whole study group; however, there was a tendency towards an inverse correlation between OGIS and osteocalcin. Furthermore, CRP and liver enzymes were comparable between GDM and CON.

### Postpartum Examination

12 weeks after delivery CTX as well as osteopontin plasma concentrations were significantly increased compared to pregnancy (ΔCTX: 0.32±0.27 ng/ml, ΔOPN: 38.50±24.33 ng/ml; both p<.0001) and did not differ between pGDM (CTX: 0.65±0.25 ng/ml, OPN: 66.82±22.0 ng/ml) and pCON (CTX: 0.67±0.29 ng/ml, OPN: 80.9±17.7 ng/ml). At postpartum visit, there was a tendency towards a positive correlation between CTX and osteocalcin (R = 0.4, p = 0.05), but no association between CTX and insulin sensitivity (OGIS) in the whole study group.

## Discussion

The current study delivers data on CTX and osteopontin in gestational diabetes, a frequent complication during pregnancy and a well known risk factor for the development of later type 2 diabetes. Furthermore, it displays a model of transiently disturbed glucose metabolism in a young female cohort. Thus we aimed to translate previous findings in basic research into a clinical investigation in the setting of GDM in order to deliver new insights in the interplay between bone and glucose metabolism.

CTX- a marker for enhanced osteoclast activity- was found elevated in women with GDM compared to healthy controls during pregnancy, with comparable concentrations postpartum. In addition, CTX was tightly associated with osteocalcin plasma concentrations as well as parameters of insulin secretion (even when grouped analyses were performed), confirming our hypothesis that changes in osteocalcin are accompanied by changes in CTX. It has been previously shown [6], that insulin signaling in the osteoblasts promotes bone resorption and acidification of the bone extracellular matrix (ECM) – via suppression of osteoprotegerin-expression-which results in activation of osteocalcin. Hence, hyperinsulinemia in gestational diabetes might increase insulin signaling in osteoblasts resulting in increased bone resorption and activation of osteocalcin, which in turn, might be able to improve insulin secretion. In our previous work, we could not state whether osteocalcin increases insulin secretion or – vice versa - insulin secretion increases osteocalcin. According to our current data and in consideration of recent findings in animal models, we hypothesize that hyperinsulinemia – via increased insulin signaling in osteoblasts- activates osteocalcin which in turn stimulates insulin secretion in pancreatic beta-cells. Although we can just hypothesize these conclusions from our current data, we can at least conclude that hyperinsulinemia during pregnancy parallels changes in CTX and osteocalcin.

It has been clearly shown, that osteocalcin ameliorates insulin sensitivity in experimental animal [4], and also studies in humans have shown a tight association between osteocalcin and improved insulin sensitivity [19], [20]. Neither osteocalcin nor CTX were positively correlated with insulin sensitivity in our study population. At least, there was a negative association between insulin sensitivity and CTX, which might be explained by the higher CTX-levels in the (more insulin-resistant) GDM-group. However, since insulin resistance during pregnancy has been identified as a physiological pattern in order to supply enough nutrients to the fetus, it might be unaffected by the positive actions of osteocalcin. In addition, the development of insulin resistance is clearly associated with hormonal changes during pregnancy. Thus, it might just in part be comparable to insulin resistance observed in obesity and type 2 diabetes.

We furthermore observed an inverse association between HDL-cholesterol and CTX in the GDM group. It has been shown that dyslipidemia parallels increased bone turnover [21]–[23]. Although these studies were performed in older subjects, this relation between bone and lipid metabolism might already exist in this young female cohort. Nevertheless, since pregnant women often show a transient impairment in their lipid profile, which normalizes after pregnancy, further studies are needed to clarify a pathological impact of these findings. CTX was increased postpartum compared to pregnancy, which is in line with prior reports that bone resorption increases during pregnancy and remains increased over lactation [24].

In contrast to CTX, osteopontin plasma concentrations were decreased in GDM compared to CON and not related to parameters of insulin secretion nor sensitivity. Hence, we assume that osteopontin-mediated subclinical inflammation underlying insulin resistance is not associated with the development of insulin resistance in GDM. However, since osteopontin action is thought to be primarily of local importance and we assessed only plasma concentrations, we cannot rule out that it might play a role in aggravated insulin resistance in obese pregnant women with GDM. Osteopontin was found to be associated with C-reactive protein as well as high-sensitive CRP which is in line with previous findings that established osteopontin as an inflammatory molecule [7]. Notably, CRP and high-sensitive CRP did not differ between GDM and healthy controls. This underlines our assumption that - in contrast to obesity-systemic low-grade inflammation does not relate to insulin resistance in GDM.

The observed association between osteopontin plasma concentrations and liver enzymes (ALT, AST) is of interest, since osteopontin was linked to the development of hepatic steatosis in obesity [25]. Furthermore, targeting osteopontin in obesity was shown to prevent the development of hepatic steatosis in animal models [26]. Thus, the observed correlation between osteopontin and liver transaminases might indicate the existence of (mild) hepatic steatosis in our young female population, independent of glucose tolerance during pregnancy. In addition, circulating osteopontin was also correlated with female sex hormones. Of note, estrogen was reported to increase hepato-biliary osteopontin expression [27]. Moreover, interactions between estrogen and osteopontin have been described in other tissues as well [28].

At least we have to point out, that due to the limitation of invasive methods during pregnancy our current findings and interpretations are simply based on plasma concentrations of CTX and osteopontin. Thus, our conclusions might serve as hypothesis for further investigations. However, the aim of this study was to translate current findings in basic research into a clinical setting of a population serving as a model of early type 2 diabetes. Given that the major finding of our study was, that hyperinsulinemia parallels CTX and osteocalcin concentrations, our data increase evidence on the similarities between mice and human concerning the interaction between bone and glucose metabolism. This is of interest, since it has been recently shown that daily injections of osteocalcin can improve glucose tolerance by increasing insulin secretion and beta cell mass and thus prevent the development of type 2 diabetes in experimental animals [29]. Hence, osteocalcin might erase as a new therapeutic option in preventing or treating type 2 diabetes.

In summary, the current study delivers evidence that CTX is higher in women with gestational diabetes compared to healthy controls and related to parameters of insulin secretion and osteocalcin. Hence, we assume that hyperinsulinemia in gestational diabetes might be related to increased bone turn/remodeling and that these interaction between bone and glucose metabolism might play a pivotal role in glucose intolerant-states. In contrast to obesity, osteopontin-mediated subclinical inflammation does not appear to underlie the development of insulin resistance in GDM.
